# Bovine Grafting: An Effective Alternative after Curettage of Benign Bone Tumors

**DOI:** 10.3390/life13030789

**Published:** 2023-03-15

**Authors:** Priscilla Montanhini, Bruno P. Antunes, Julie Francine Cerutti Pestilho, Carlos Roberto Galia, Alex Guedes, Ricardo Gehrke Becker

**Affiliations:** 1 Hospital de Clínicas de Porto Alegre (HCPA), Rua Ramiro Barcelos, 2350, Porto Alegre 90035-903, RS, Brazil; 2 Hospital Moinhos de Vento (HMV), Rua Ramiro Barcelos, 910, Porto Alegre 90035-000, RS, Brazil; 3 Instituto do Câncer Infantil do Rio Grande do Sul, Rua São Manoel, 850, Porto Alegre 90620-110, RS, Brazil; 4 Hospital Santa Izabel, Praça Conselheiro Almeida Couto, 500, Salvador 40050-410, BA, Brazil

**Keywords:** bone grafting, biocompatible materials, bone substitute, xenografts, bone neoplasms, bone cysts

## Abstract

We retrospectively reviewed 28 patients (15 women and 13 men) with benign bone tumors or pseudotumors treated with curettage and filling with freeze-dried bovine bone graft Orthogen (Baumer S/A, São Paulo, Brazil). The aim of the study was to evaluate the rate of incorporation of Orthogen into the host bone, as well as to describe the outcomes of bone healing (quality, time, and complications). General characteristics, tumor volume, size, site, complications, percent filled, and healing quality at 6 and 12 months were assessed through radiographs. Mean patient age was 20.5 (range 4.7–75.1) years. The most common lesion type was simple bone cyst (12/28), and the most common sites were the tibia (7/28) and humerus (7/28). There were no postoperative pathologic fractures. Two cases (7.1%) of serous fluid leakage through the wound occurred. Mean cavity volume was 20.1 (range 2.7–101.4) cm^3^. At 6 and 12 months, 75% and 77.8% of cavities, respectively, showed complete bone healing. At 12 months, 81% of cavities filled >90% with graft showed complete bone healing vs. only 19% of those filled <90%. Filling with bovine bone graft resulted in few complications and excellent healing after curettage of benign bone tumors or pseudotumors. Complete healing occurred in most cases by 12 months. Cavities with a higher percentage of filling had a higher rate of complete radiographic incorporation.

## 1. Introduction

In recent decades, bone substitutes have become increasingly common in orthopedic and dental surgery. There has also been an increase in heterologous bone grafts, called xenografts, which are derived from bovine, porcine, coral, crustacean, or sericultural sources [1,2,3]. Among them, bovine bone grafts have been the most common type due to their physical and chemical similarity to human bone. In addition, they are widely available in the market, have a long storage time, and are easy to handle in the operating theater. Some authors have expressed concern about antigenic potential and contamination by prions, but preparation and manufacture of this type of graft on an industrial scale have mitigated these risks [4,5,6,7,8].

Bovine bone grafts can be distributed as deproteinized, maintaining the inorganic phase of the natural bone, or demineralized, maintaining the organic phase of the natural bone, or partially deproteinized. The final product is obtained by physical–chemical processing, aiming to maintain bone structure, reduce immunogenicity, and provide a favorable environment for cell adhesion and new bone formation. The biological characteristics of bovine bone grafts make this biomaterial suitable for filling bone cavities made by curettage of benign bone tumors or pseudotumors [3,9].

Several studies in orthopedics and biomaterial science have described the biocompatibility, the histological characteristics of the newly formed bone after implantation, the compressive strength of bovine bone grafts, and their successful use in hip arthroplasties, knee osteotomies, and foot arthrodesis [4,6,7,10,11,12,13,14,15]. We, therefore, reviewed a series of patients with benign bone tumors or pseudotumors treated with curettage, a local adjuvant (when necessary), and filling with freeze-dried bovine bone graft. We aimed to evaluate whether Orthogen bovine bone graft presents good rates of incorporation into the host bone after curettage of benign bone tumors, describe the radiographic healing characteristics after cavity filling, and assess postoperative complications.

## 2. Materials and Methods

This study was approved by the institutional research ethics committee, and all participants or their legal guardians provided written informed consent prior to inclusion.

**Figure 1:**
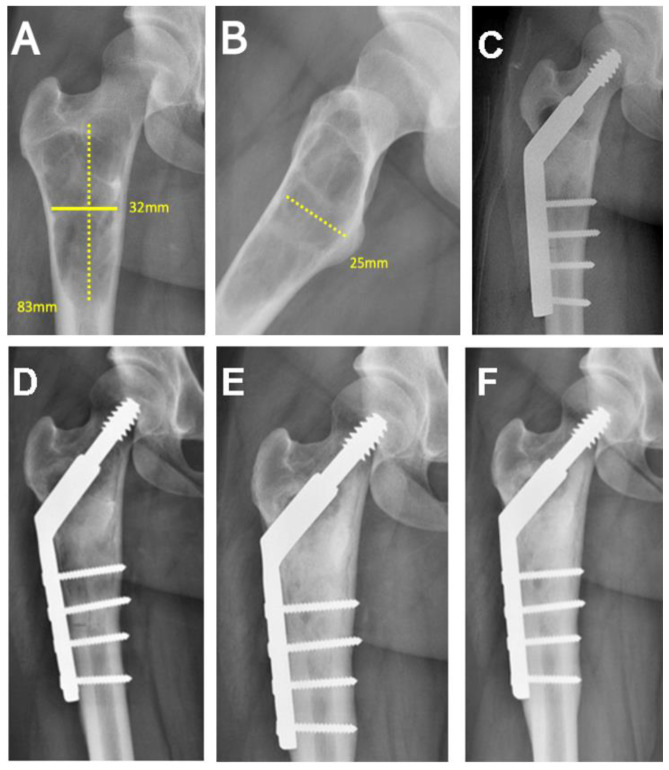
(**A**,**B**) Preoperative radiograph, anteroposterior, and lateral views. Volume measurement in a unicameral bone cyst of the proximal femur. Application of the volume formula ABC × 0.785 = 52.25 cm^3^ (cylindrical defect). (**C**) Immediate postoperative radiograph after curettage, grafting, and plate fixation. Bone graft homogeneously distributed in the cavity with >90% filling. (**D**) Postoperative 6-month follow-up radiograph showing cortical thickening and partial graft incorporation. (**E**,**F**) Radiographs at 12- and 24-month follow-up, showing complete graft incorporation (Neer I) and bone remodeling.

**Figure 2:**
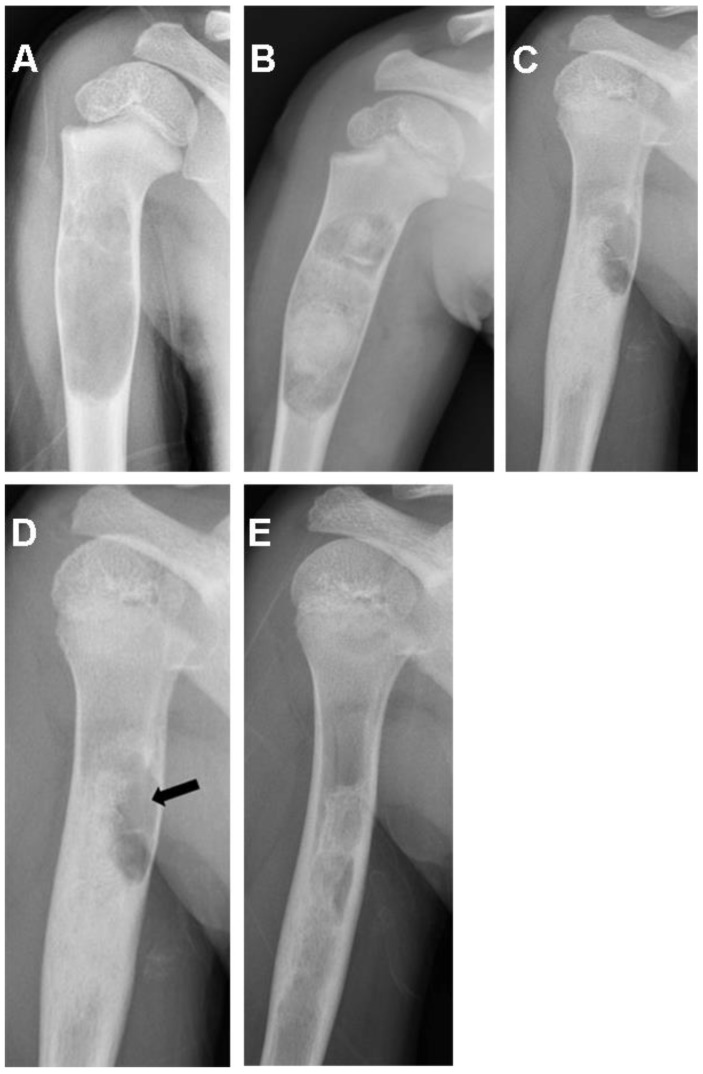
(**A**) Preoperative radiograph of a unicameral bone cyst of the humerus. (**B**) Immediate postoperative radiograph after curettage and bone graft filling <90% of the cavity. (**C**,**D**) Postoperative 6- and 12-month follow-up radiographs showing radiolucent area <50% (arrow) of the bone diameter (Neer II). (**E**) Twenty-four-month follow-up radiograph showing cortical thickening, bone remodeling, and small intramedullary cystic remnants.

**Figure 3:**
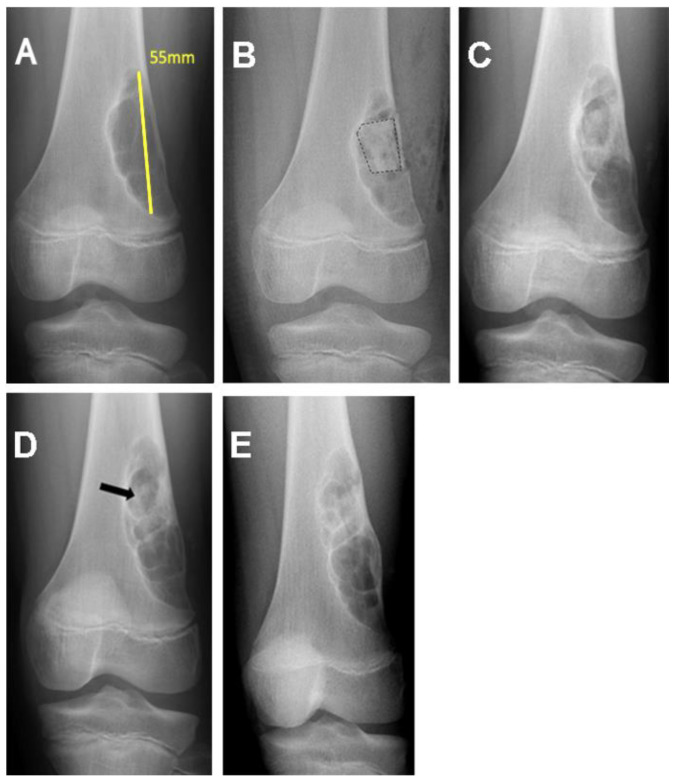
(**A**) Preoperative radiograph of a nonossifying fibroma of the distal femur. (**B**) Immediate postoperative radiograph after curettage showing poor cavity filling with bone graft. Dashed lines delimit the grafted area. (**C**) Postoperative 6-month follow-up radiograph showing persistent radiolucent areas > 50% of the bone diameter (Neer III) and (**D**) 12-month follow-up radiograph with remnants of the graft in the proximal region of the cavity (arrow). (**E**) Twenty-four-month follow-up radiograph showing persistent cystic areas and complete resorption of the bone graft.

The medical records of 28 patients with benign nonaggressive bone tumors or pseudotumors treated consecutively by 2 orthopedic surgeons (RGB and BPA) through curettage and filling with freeze-dried bovine bone graft were reviewed. Other types of bone grafts (autograft, synthetic hydroxyapatite, or no filling) were not included in this sample; as well, there were no case controls due to the small number of patients. The variables extracted from the medical records were the volume (cm^3^) and size (cm) of the tumor, the number of graft units used, anatomical site, histological diagnosis, percent of the cavity filled, and quality of healing assessed radiographically, as described below. Preoperative and immediate postoperative radiographs of the bone lesions were digitally measured in Enterprise Imaging 8.1.2 SP7.1 (Agfa HealthCare, Mortsel, Belgium) (Figure 1A–C, Figure 2A,B and Figure 3A,B). The volumes of cylindrical and spherical cavities were calculated, respectively, using the formulas ABC × 0.785 and ABC × 0.52 (A = width, B = depth, and C = height). Immediate postoperative cavity filling was measured as >90% or <90%, with > 90% as the treatment goal (Figure 1C, Figure 2B and Figure 3B). Cavities filled with <90% occurred due to limited amount of graft available (1 patient) or poor impaction (7 patients). After surgery, radiographs were taken at 6, 12, and 24 months to assess graft healing (Figure 1D–F, Figure 2C–E and Figure 3C–E). The quality of graft healing was evaluated using a modified Neer classification for bone cysts. The classification is based on 4 categories: I—healed cavity filled with new bone, with radiolucent areas <10 mm; II—healed with radiolucent areas < 50% of the bone diameter; III—persistent radiolucent areas >50% of the bone diameter; and IV—recurrent cavity in a previously healed area [16]. The Neer classification was chosen because of the similar behavior of tumors in our sample with simple bone cysts.

**Figure 4:**
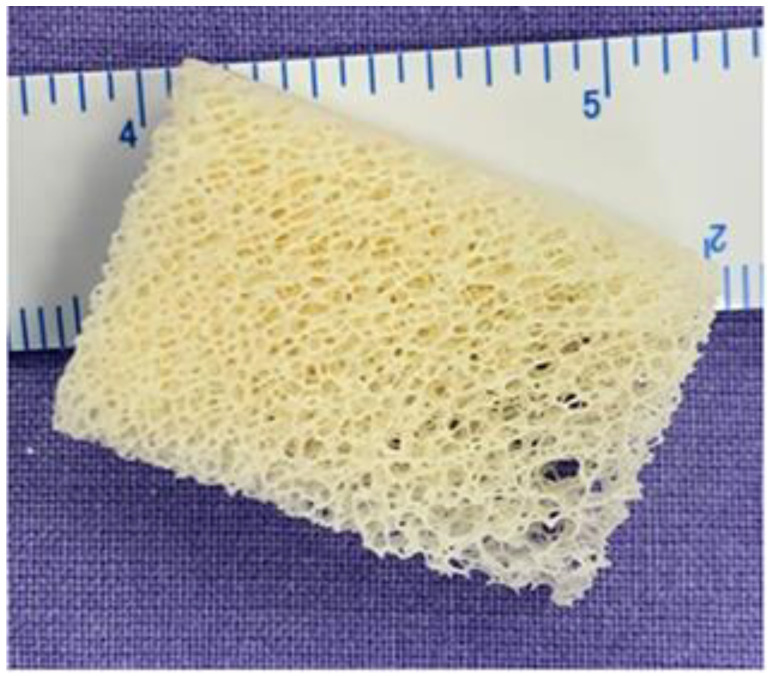
Orthogen bone graft block (10 × 20 × 30 mm).

Pseudotumor lesions were curetted and grafted, whereas benign neoplasms received intraoperative adjuvant treatment (drilling, fulguration, or ethanol) before grafting. Lesions that presented with a pathologic fracture were filled through the fracture before surgical reduction and fixed with orthopedic implants. In cases of imminent fracture, curettage was performed, the lesions filled by opening a bone window, and the periosteum was opposed. All lesions were filled with Orthogen (Baumer S/A, São Paulo, Brazil) bovine bone graft, which has a mixed structure composed of an organic portion (25–30% collagenous proteins) and a mineral portion (65–70% hydroxyapatite) (Figure 4). The 10 × 20 × 30 mm blocks were hydrated, chopped, and mechanically compacted in the cavity created by the curettage. No autologous bone graft or bone marrow aspirate was added to the bovine graft. All caution was taken to avoid leaving remnants of bone graft on the soft tissues.

Quantitative variables were described as mean and standard deviation (SD) or median and interquartile range (IQR). Qualitative variables were described as absolute and relative frequencies.

## 3. Results

The patient and tumor characteristics are summarized in Table 1. A total of 15 women and 13 men with a mean age of 20.5 (range 4.7–75.1) years were followed up for a minimum and maximum of 8 and 30 months, respectively. The most common lesions were simple bone cysts (12/28), cartilaginous tumors (5/28), osteofibrous dysplasia (4/28), and aneurysmal bone cysts (3/28). Most were located in the tibia (7/78), the proximal metaphyseal segment of the humerus (7/28), or the distal (4/28) and proximal (4/28) femur. The mean lesion volume was 21.0 (range 2.7–101.4) cm^3^.

Healing quality was assessed through radiographs using the Neer classification system. At 6 months, 21 of 28 patients (75%) were classified as Neer I, 5 (17.9%) as Neer II, 2 (7.1%) as Neer III, and 0 as Neer IV. A total of 27 patients completed 12 months of follow-up, and all of them attained the quality assessed at 6 months (Table 2). At the 12-month radiographic evaluation, complete healing occurred in 81% (17/21) of patients when >90% of the cavity was filled. Conversely, complete healing occurred in only 19% (4/21) when <90% of the cavity was filled (Table 3).

Two patients (7%) had complications within 30 days postoperatively, both of whom had serous drainage between days 6 and 12 with mild hyperemia that was resolved through dressings and oral antibiotics (cephalosporin) before postoperative day 21. Local recurrence occurred in two patients (7%) after 24 and 36 months postoperatively (case numbers 17 and 3). Both were resolved after curettage and regrafting with Orthogen. No pathologic fractures occurred postoperatively.

## 4. Discussion

Applying bone substitutes after curettage has shown lower rates of postoperative fractures than unfilled cavities [17]. Curettage allows the bone to slowly regain its original strength, and the substitute, according to its intrinsic characteristics, provides stiffness and accelerates healing. In a systematic review of 2555 patients, Gava et al. [17] found that the fracture prevalence after curettage was 6.6% in unfilled cavities, 2.1% after allograft, 2.0% after bone substitutes, 1.7% after autograft, and 0% after xenograft. Although the results favored cavity filling, there was no statistical correlation between graft type and healing time [17].

Currently used biomaterials include synthetic bone substitutes (hydroxyapatite, as beta-tricalcium phosphate ceramics, calcium sulfate, polymers, bioactive glass, and composites), autografts, allografts, xenografts (bovine, chitosan, and silk), cement, and bone substitutes with growth factors [9]. The advantages and disadvantages of substitutes, as well as the characteristics of the recipient area, determine the choice of biomaterial and healing time. Autografts, for example, have the best biological characteristics and require the shortest time for bone incorporation. However, in orthopedic surgery, autograft reconstructions are limited by the graft volume and morbidity in the donor area. Likewise, allografts (industrialized or frozen) have good histocompatibility, adequate mechanical resistance, and an abundant supply. On the other hand, logistical and regulatory difficulties, immunogenicity, the risk of viral transmission, and expiration of the material can hamper their use. Similarly, although synthetic bone substitutes are widely available, they are also limited by high cost, lower osteoinductive capacity, and lower mechanical strength, depending on the material [1,8].

In the last two decades, bone xenografts have been used more frequently in orthopedic and dental surgery [18]. Several types have emerged as alternatives in the market, from bovine and porcine grafts to silk and crustaceans. In addition, different product presentations are available, such as bovine-derived organic (Orthogen, Hypro-Oss) or inorganic (GenoxInorgânico, Bio-Oss, Bonefill) bone grafts and calcium phosphate ceramics from marine corals (CoreBone, BoneMedik). Among these presentations, bovine bone grafts most closely resemble the structure of human bone. The porous architecture of bovine bone tissue, in addition to being rich in hydroxyapatite, also provides relative mechanical support and allows osteoconduction and the migration of blood vessels to the interior through neoangiogenesis [3]. Galia et al. [19] demonstrated in vitro that Orthogen bovine bone graft presented a medullary bone structure with interconnected pores and a trabecular crystal structure that favors the deposition of osteoprogenitor cells, physiological resorption, and osteoid apposition.

Bracey et al. [20] conducted a historical analysis of studies on bone xenografts in orthopedic surgery. Almost 50% of the studies were based on spinal procedures, and unfavorable results were found in 47% of all studies, leading the author to discourage the use of bone xenograft. Charalambides et al. [21] and Shibuya and Jupiter [8] have criticized bovine bone graft due to poorer graft incorporation outcomes, lower rates of spine and foot fusion, and higher rates of inflammatory reactions than autogenous bone graft. Kim et al. [22] and Laurencin and El-Amin [23] cited the concern expressed in international studies with the risk of nondetectable pathogen transmission into humans after xenograft transplantation.

On the other hand, Hugen et al. [9] conducted a review of the properties of “ideal” bone graft substitutes in craniofacial and periodontal applications. Similar incorporation outcomes to other bone sources were found after bovine bone grafting. Likewise, there were no reports of transmissible spongiform encephalopathy or bovine spongiform encephalopathy. In 2009, Rosito et al. [11] described a series of 25 patients with severe acetabular defects treated with bovine bone graft in revision total hip arthroplasty. Eighteen patients (72%) presented good or very good radiographic incorporation of the bovine bone graft with minor graft-related complications. Henning et al. [10] evaluated the rate of union after subtalar arthrodesis with autologous (6) and freeze-dried bovine bone graft (6). Solid union was achieved in all patients except one in the xenograft group.

Despite the controversy, bovine bone graft has excellent applicability in surgical practice due to its availability, acceptable incorporation rate and time, lower cost than synthetic substitutes, and long storage time [24]. Our results were similar to those of previous papers using different sources of bone grafts for bone cavities secondary to curettage [16,17,25]. According to our findings, the highest percentage of cavity filling showed a trend toward better bone graft incorporation and healing. Based on our clinical practice, the use of a meticulous impaction technique has a considerable effect on the results. Furthermore, the porosity scaffold of the bovine bone graft applied to a well-vascularized host cavity seems to corroborate our clinical outcomes. In addition, the low complication rate and excellent healing allow Orthogen bovine bone graft to be safely used after curettage of benign bone tumors and pseudotumors.

## 5. Conclusions

Orthogen bovine bone graft after curettage of benign bone tumors resulted in few complications and an excellent healing rate at 6 and 12 months. Complete bone graft incorporation occurred in most cases. Cavities filled >90% were more likely to exhibit full graft incorporation.

## Figures and Tables

**Table 1:** Patient and tumor characteristics.

Patient Registry/Sex/Age (Years)	Tumor Location	Pathological Diagnosis	Tumor Volume (cm^3^)
1/F/14	Calcaneus	Simple bone cyst	5.82
2/F/8	Humerus	Simple bone cyst	4.12
3/M/5	Proximal femur	Simple bone cyst	7.05
4/F/28	Calcaneus	Simple bone cyst	8.53
5/M/75	Proximal tibia	Ganglion cyst	21.84
6/M/7	Distal femur	Simple bone cyst	62.96
7/M/45	Phalanx (foot)	Gouty tophi	8.24
8/F/40	Phalanx (foot)	Enchondroma	4.71
9/M/17	Humerus	Fibrous dysplasia	16.28
10/F/32	Phalanx (hand)	Epithelial bone cyst	8.24
11/F/10	Tibia	Nonossifying fibroma	23.55
12/F/11	Tibia	Chondromyxoid fibroma	14.87
13/F/29	Proximal femur	Simple bone cyst	7.63
14/M/9	Tibia	Aneurysmal bone cyst	26.82
15/F/9	Humerus	Simple bone cyst	70.2
16/M/5	Humerus	Aneurysmal bone cyst	3.6
17/M/11	Distal femur	Nonossifying fibroma	7.11
18/M/16	Fibula	Chondromyxoid fibroma	27.66
19/F/15	Proximal femur	Simple bone cyst	52.25
20/M/4	Humerus	Simple bone cyst	14.49
21/F/4	Tibia	Aneurysmal bone cyst	12.35
22/F/12	Humerus	Simple bone cyst	8.22
23/F/8	Tibia	Nonossifying fibroma	9.36
24/M/38	Proximal femur	Simple bone cyst	101.42
25/F/24	Pelvis	Ganglion cyst	2.73
26/F/24	Distal femur	Chondroblastoma	5.61
27/M/9	Humerus	Simple bone cyst	37.28
28/M/46	Distal femur	Enchondroma	14.62

**Table 2:** Radiographic assessment status at 6, 12, and 24 months after curettage and grafting.

Radiological Evaluation	6 Months *n* (%)	12 Months *n* (%)	24 Months *n* (%)
	(*n* = 28)	(*n* = 27)	(*n* = 16)
Neer * I (healed cavity)	21 (75.0)	21 (77.8)	12 (75.0)
Neer II (healed with defects)	5 (17.9)	5 (18.5)	1 (6.3)
Neer III (persistent lesion)	2 (7.1)	1 (3.7)	3 (18.8)
Neer IV (recurring lesion)	0 (0.0)	0 (0.0)	0 (0.0)

* Neer classification for healing status.

**Table 3:** Healing status and cavity filling percentage at 12-month follow-up.

	(Neer I)	(Neer II, III, IV)
	*n* (%)	*n* (%)
Percentage of cavity filled		
<90%	4 (19.0)	4 (66.7)
>90%	17 (81.0)	2 (33.3)

## Data Availability

All relevant data are within the paper.
